# Increase in Methadone Treatment Episodes With Co-occurring Mental Health Disorders between 2013 and 2023

**DOI:** 10.21203/rs.3.rs-10654615/v1

**Published:** 2026-10-05

**Authors:** Yinfei Kong, Kechen Zhao, Alanis Goncalves Leite, Daniel Howard

**Affiliations:** California State University, Fullerton; AstraZeneca Foundation; Texas A&M University; Texas A&M University

## Abstract

**Objectives:**

To compare US methadone treatment episodes with co-occurring mental health disorders in 2013 and 2023.

**Methods:**

We analyzed 2013 and 2023 Treatment Episode Data Set-Discharges records for 75,338 episodes with methadone use, a co-occurring mental health disorder, valid sex or gender information, and discharge because treatment was completed, dropped out, or terminated by the facility. We compared demographics, substance use, treatment, and discharge characteristics.

**Results:**

Eligible episodes increased from 18,935 in 2013 to 56,403 in 2023. Compared with 2013, 2023 episodes involved older clients (mean age, 39.72 vs 36.16 years), fewer female clients (40.2% vs 49.8%), more methamphetamine/speed use (14.7% vs 3.2%), more cocaine/crack use (38.8% vs 29.9%), less injection drug use (37.1% vs 55.3%), more Medicaid payment (22.9% vs 7.7%), and more homelessness (19.3% vs 12.7%).

**Conclusions:**

The profile of methadone treatment episodes with psychiatric comorbidity changed markedly.

## Introduction

The US opioid overdose epidemic has changed substantially over the past decade, moving from a period dominated by prescription opioid and heroin use toward a polysubstance epidemic in which illicitly manufactured fentanyl, stimulants, and other substances frequently co-occur.^1–4^ These changes have altered the needs of patients entering opioid use disorder treatment.

Co-occurring mental health disorders are common among patients with opioid use disorder and are associated with greater clinical complexity and adverse outcomes.^5–8^ For treatment programs, psychiatric comorbidity can affect medication management, behavioral health staffing, discharge planning, and coordination with housing and social services.

Methadone remains a central medication for opioid use disorder, particularly for patients with high treatment acuity and complex social needs. Major policy and service changes, including Medicaid expansion and evolving treatment delivery models, may have changed the profile of patients receiving methadone treatment.^10^ We compared publicly funded methadone treatment episodes with co-occurring mental health disorders in 2013 and 2023.

## Methods

We used the 2013 and 2023 Treatment Episode Data Set-Discharges, a national episode-level administrative data system maintained by the Substance Abuse and Mental Health Services Administration.^11,12^ TEDS-D includes discharge records from publicly funded substance use treatment facilities in the United States. The analysis used public, deidentified data; trial registration was not applicable.

For each year, the analytic sample was restricted to discharge episodes with methadone use reported, a co-occurring mental health disorder present, valid sex or gender information, and a discharge reason of treatment completed, dropped out, or terminated by facility. These criteria yielded 18,935 episodes in 2013 and 56,403 episodes in 2023.

Variables were harmonized across years to create comparable categories for race and ethnicity, education, employment, living arrangement, arrests, prior treatment episodes, service setting, region, age at first use of the primary substance, pregnancy, veteran status, and Medicaid as primary payment source. Race and ethnicity were assessed to describe equity-relevant differences in the treatment population. Hispanic or Latino ethnicity was prioritized over race.

Length of stay was converted to approximate days using TEDS-D category midpoints. We compared years using independent-samples t tests for continuous variables and chi-square tests for categorical and binary variables. P values were 2-sided and descriptive.

## Results

The sample included 75,338 methadone treatment episodes with co-occurring mental health disorders: 18,935 in 2013 and 56,403 in 2023, representing a 198% increase in eligible discharge episodes (Table 1). Mean age at admission increased from 36.16 years in 2013 to 39.72 years in 2023. The proportion of episodes involving female clients decreased from 49.8% to 40.2%.

Substance use patterns changed substantially. Methamphetamine/speed was reported at admission in 14.7% of 2023 episodes compared with 3.2% of 2013 episodes. Cocaine/crack reporting increased from 29.9% to 38.8%, and alcohol reporting increased from 18.1% to 26.0%. Current injection drug use was reported less often in 2023 than in 2013 (37.1% vs 55.3%).

Treatment and discharge characteristics also shifted. Medicaid as the primary payment source increased from 7.7% to 22.9%. Ambulatory treatment accounted for a smaller share of episodes (59.2% vs 73.7%), while rehabilitation or residential services accounted for a larger share (30.1% vs 9.2%).Treatment completion increased from 31.0% to 41.5%, and homelessness at admission increased from 12.7% to 19.3%.

## Discussion

In this repeated cross-sectional analysis, the national profile of publicly funded methadone treatment episodes with co-occurring mental health disorders changed markedly between 2013 and 2023. The eligible population grew by nearly 200%, became older on average, included a lower proportion of female clients, and showed greater reporting of stimulant co-use, Medicaid payment, residential or rehabilitation services, treatment completion, and homelessness.

The increase in methamphetamine/speed reporting is especially important for treatment planning. Unlike opioid use disorder, stimulant use disorder has no medication with evidence comparable to methadone, buprenorphine, or naltrexone for opioid use disorder. Programs treating patients with both opioid use disorder and mental health disorders may therefore need stronger capacity for contingency management, integrated psychiatric care, harm reduction counseling, and management of stimulant-associated psychiatric symptoms.^9^

The increase in Medicaid as primary payment source is consistent with broader changes in insurance coverage and behavioral health financing after the Affordable Care Act.^10^ Because many patients with opioid use disorder and psychiatric comorbidity rely on public insurance, Medicaid policy may be closely tied to access to methadone treatment, integrated behavioral health services, and transitions after discharge. The concurrent increase in homelessness suggests that clinical services alone may be insufficient without housing and social service linkages.

This study has limitations. TEDS-D records treatment episodes rather than unique individuals. The co-occurring mental health disorder indicator does not specify diagnosis, severity, treatment receipt, or timing. TEDS-D includes publicly funded treatment facilities and may not represent all private or office-based treatment. State reporting practices and variable definitions may have changed despite harmonization. Analyses were descriptive and unadjusted; observed differences should be interpreted as associations with calendar year rather than effects of policy or clinical change.

## Figures and Tables

**Table 1:** Characteristics of Methadone Treatment Episodes With Co-occurring Mental Health Disorders, 2013 and 2023

Characteristic	2013 (N = 18,935)	2023 (N = 56,403)	P value
Demographics			
Female sex	9,423 (49.8)	22,667 (40.2)	< .001
Race or ethnicity			< .001
White	13,426 (70.9)	38,005 (67.4)	
African American	2,228 (11.8)	6,921 (12.3)	
Latino	2,581 (13.6)	8,205 (14.5)	
Other	529 (2.8)	2,459 (4.4)	
Unknown	171 (0.9)	813 (1.4)	
Age at admission, y	36.16 (10.98)	39.72 (10.99)	< .001
Substance use at admission			
Current injection drug use	10,476 (55.3)	20,911 (37.1)	< .001
Alcohol use	3,418 (18.1)	14,691 (26.0)	< .001
Cocaine or crack use	5,660 (29.9)	21,860 (38.8)	< .001
Marijuana or hashish use	3,553 (18.8)	9,859 (17.5)	< .001
Methamphetamine or speed use	612 (3.2)	8,268 (14.7)	< .001
Treatment characteristics			
Treatment setting			< .001
Detoxification	3,229 (17.1)	5,999 (10.6)	
Rehabilitation/residential	1,750 (9.2)	16,994 (30.1)	
Ambulatory	13,942 (73.7)	33,410 (59.2)	
Medicaid primary payment source	1,452 (7.7)	12,926 (22.9)	< .001
One or more prior treatment episodes	16,046 (84.7)	48,196 (85.4)	.048
Treatment, discharge, and living characteristics			
Length of stay, d	148.71 (150.57)	128.04 (142.66)	< .001
Reason for discharge			< .001
Treatment completed	5,864 (31.0)	23,390 (41.5)	
Dropped out	9,391 (49.6)	27,783 (49.3)	
Terminated by facility	3,680 (19.4)	5,230 (9.3)	
Living arrangement at admission			< .001
Homeless	2,404 (12.7)	10,877 (19.3)	
Dependent living	2,030 (10.7)	8,416 (14.9)	
Independent living	14,395 (76.0)	34,188 (60.6)	
Unknown	106 (0.6)	2,922 (5.2)	

Note. Values are n (%) or mean (SD). P values are from independent-samples t tests for continuous variables and chi-square tests for categorical or binary variables; they are descriptive and were not adjusted for multiplicity. TEDS-D indicates Treatment Episode Data Set-Discharges.

## Data Availability

TEDS-D 2013 and 2023 public-use data are publicly available from the Substance Abuse and Mental Health Services Administration.

## Public Health Implications:

Treatment systems may need greater capacity for stimulant co-use, aging-related needs, Medicaid-financed integrated care, and housing support.

## Public Health Implications

Methadone treatment episodes with co-occurring mental health disorders changed between 2013 and 2023 in ways that suggest increasing needs related to stimulant co-use, aging, Medicaid-financed care, and housing instability. Treatment systems serving this population may benefit from integrated psychiatric and addiction care, services for stimulant co-use, and stronger coordination with housing and social support resources.

## Data Availability

The data that support the findings of this study are openly available in Substance Abuse and Mental Health Services Administration at https://www.samhsa.gov/data/data-we-collect/teds-treatment-episode-data-set/datafiles/teds-d-2013 and https://www.samhsa.gov/data/data-we-collect/teds-treatment-episode-data-set/datafiles/teds-d-2023.
